# Remediation of chromium- and fluoride-contaminated groundwater by immobilized Citrobacter sp. on a nano-ZrO_2_ hybrid material

**DOI:** 10.1371/journal.pone.0253496

**Published:** 2021-06-23

**Authors:** Xilin Li, Ming Fan, Ying Zhang, Ling Liu, Fu Yi, Jinghua Chang, Jian Li

**Affiliations:** 1 School of Civil Engineering, Liaoning Technical University, Xihe District, Fuxin, Liaoning Province, People’s Republic of China; 2 School of Civil and Architectural Engineering, Chuzhou University, Langya District, Chuzhou City, Anhui Province, People’s Republic of China; 3 School of Architecture and Transportation, Liaoning Technical University, Xihe District, Fuxin, Liaoning Province, People’s Republic of China; 4 School of Science, Liaoning Technical University, Xihe District, Fuxin, Liaoning Province, People’s Republic of China; University of Southern Denmark, DENMARK

## Abstract

To effectively address excessive SO_4_^2-^, Cr(VI), total chromium and F^-^ in the groundwater of acidic mining areas, a facultative anaerobic bacterium, Citrobacter, with sulfate-reducing properties, tolerance to hexavalent chromium and the ability to reduce Cr(VI) to Cr(III) was isolated and domesticated. Based on microbial immobilization technology, a nano-ZrO_2_ polyacrylamide hybrid material was prepared as an embedding agent to form nano-ZrO_2_ polyacrylamide Citrobacter (ZPC) particles. ZPC was microscopically characterized, and the removal performance and mechanism of ZPC for SO_4_^2-^, Cr(VI), total chromium and F^-^ in groundwater were analyzed. The results of single-factor tests showed that the optimal reaction conditions included a reaction temperature of 35°C, Citrobacter dosage of 35% (volume ratio) in the particles and hybrid material dosage of 300 mL; under these conditions, the removal rates of SO_4_^2-^, Cr(VI), total chromium and F^-^ were 70.5%, 100%, 100% and 93.3%, respectively, and the pH value increased from 4.6 to 8.07. On this basis, the effects of the reaction layer type, influent hydraulic load and influent concentration on the removal efficiency of polluted groundwater were studied through dynamic experiments. The experimental results showed that ZPC particles were better than Citrobacter as a reaction layer; the optimal influent hydraulic load was 3.0 m^3^/(m^2^·d); the selectivity of ZPC particles to anions and anionic groups was different; and the order of adsorption selectivity was F^-^ > Cr(VI) > SO_4_^2-^.

## Introduction

Many industrial enterprises, such as those related to mining, metallurgy, petrochemicals, machinery, electronics, medicine, electroplating, leather, pigments, pesticides and semiconductors, produce large amounts of fluorine- and chromium-containing industrial wastewater, sludge, and solid waste residues in the production process [1]. If handled improperly, heavy metal ions such as Cr(VI) and Cr(III) and inorganic anions such as F^-^ and SO_4_^2-^ will cause serious groundwater pollution [2]. Drinking groundwater containing fluorine and chromium will seriously affect human health. For example, the excessive intake of fluoride can lead to dental fluorosis, skeletal fluorosis, and some nervous system diseases [3]. Chromium, one of the three carcinogenic metals recognized in the world, seriously affects the metabolism and other physiological activities of humans and other organisms [4,5]. Therefore, it is of great practical significance and far-reaching historical significance to remediate groundwater contaminated by fluorine and chromium.

Biological treatment of heavy metal-contaminated groundwater has the advantages of a low treatment cost, the ability to treat many kinds of pollutants and no secondary pollution, and it has become increasingly popular at home and abroad [6,7]. Most studies on biological methods are based on the dissimilation of sulfate-reducing bacteria (SRB) to reduce SO_4_^2-^ to S^2-^, as S^2-^ and metal ions in wastewater produce sulfide precipitation [8]. In the past 20 years, more than 33 types of SRB have been identified [9]. The treatment of heavy metal ions with SRB has shown excellent characteristics in the laboratory. However, SRB are almost exclusively strict anaerobic bacteria. When SRB are used in practical engineering applications, they exhibit poor adaptability to complex oxygen environments and have limited removal effects. The research and development of facultative anaerobes with reducing ability has become a topic of extensive interest for scholars [10]. Srinath isolated 8 high-chromate-resistant facultative anaerobes from tanning wastewater, which could reduce 400 mg/mL Cr(VI) by 70% [11]. Cui et al. isolated the chromium-tolerant facultative anaerobic species *Bacillus shackletonii* from heavy metal-contaminated soil. The minimum inhibitory concentration of Cr(VI) was 600 mg/L [12]. The citric acid bacteria studied by Qiu could completely reduce 10 mM sulfate to sulfide within 7 days and effectively precipitate copper ions. After 7 days of aerobic growth, the sulfate reduction capacity was restored [13]. Zhang isolated a new nontraditional species of SRB from anaerobic sludge beds and identified it as *Citrobacter freundii*. The removal efficiencies of thallium (Tl) and sulfate from acid mine drainage reached 99.60% and 89.80%, respectively [14]. Wang isolated and purified a Citrobacter strain from soil near a gold mine. In solutions with initial copper ion concentrations of 0.5 mmol/L and 1 mmol/L, the best copper ion removal effect was achieved when the reaction lasted for 120 h, with removal rates of 69% and 70%, respectively [15]. Based on these results, the research team isolated and purified facultative anaerobic bacteria from the activated sludge of a leather industrial park. The strain was Citrobacter with a sulfate reduction function and was used to remove pollutants such as SO_4_^2-^, Cr(VI), Cr(III) and F^-^ in mine groundwater.

Although many excellent strains with potential application prospects in water treatment have been screened, the application of microbial technology in practical engineering is still limited by some factors. Due to the complex composition of polluted groundwater, it is difficult to reach standard limit values by a single treatment technology. Most studies are limited to the use of microorganisms to treat heavy metal pollution in water, and the effective treatment of water that contains both heavy metal and fluoride ion pollution cannot be achieved. Therefore, in view of the trend of complex water pollution, it is necessary to prepare water treatment adsorbents that can simultaneously treat heavy metal ions and multiple inorganic anions. Embedding and immobilization can allow microorganisms to maintain a high cell density and low cell activity loss in a water environment [16]. The choice of embedding material is an important factor that affects microbial immobilization. On the one hand, the ideal material should have the characteristics of low cost and easy availability, nontoxicity to microorganisms, nonbiodegradability, good mass transfer performance, sufficient living space for microorganisms, and easy treatment and regeneration [17]. On the other hand, the material should also be usable as an adsorbent in combination with embedded microorganisms to remove toxic and harmful substances in water [18]. It was found that adsorbents with zirconium as the carrier showed good performance in removing harmful inorganic anions from wastewater [19,20]. Zirconia is widely used because of its simple production process, low cost, stable chemical properties, and long-term existence under harsh conditions such as acidic and alkaline conditions. Moreover, zirconia itself is a special amphoteric oxide and has a certain reduction capacity, and thus, it is widely used [21,22]. Nanozirconia can be crosslinked with organic compounds to form organic/inorganic hybrid materials [23,24]. Ahmad [25] synthesized an organic/inorganic composite material, polyacrylamide sulfonyl zirconium (IV), by sol-gel technology and found that the material has a fairly high ion exchange capacity for lead ions. Some scholars have found that zirconium ions can be directly crosslinked with polyacrylamide to synthesize colloidal particles of hybrid materials [26]. However, the particles are not well tolerated and are unstable under acidic or alkaline conditions. Gel particles prepared from acrylamide monomers have good resistance to temperature and salinity [27].

Our hypothesis was that Citrobacter would exhibit a strong reducing ability for Cr(VI) and sulfate, while the nano-ZrO_2_ polyacrylamide hybrid material would exhibit a high adsorption ability towards F^-^, Cr(Ⅲ) and Cr(VI). Nano-ZrO_2_ polyacrylamide Citrobacter (ZPC) formed by embedding would simultaneously remove F^-^, Cr(VI), Cr(Ⅲ) and sulfate [28]. This study used a nano-ZrO_2_-polyacrylamide hybrid material obtained by the hybrid polymerization of ZrOCl_2_ and acrylamide monomer, which was used as an embedding agent to immobilize Citrobacter to form ZPC. On this basis, ZPC was microscopically characterized, the remediation of chromium- and fluorine-polluted groundwater by ZPC particles was studied, and the best remediation conditions were determined through static and dynamic tests. The results provide reference data for engineering applications.

## Materials and methods

### Test materials

#### Source of strain

The sludge used for the strain was taken from activated sludge in a leather industrial park in Fuxin City, Liaoning Province, China. The permit for taking activated sludge samples was permitted by Fuxin Municipal Ecology and Environment Bureau.

#### Medium

1. Enrichment medium: KNO_3_ (1 g/L), Na_2_HPO_4_ (0.5 g/L), MgSO_4_·7H_2_O (0.6 g/L), CaSO_4_·2H_2_O (0.5 g/L), FeSO_4_·7H_2_O (0.5 g/L), peptone (1 g/L), yeast extract (1 g/L), and sodium citrate (3 g/L) were mixed in distilled water (1 L) at pH = 8 and sterilized at 121°C for 20 mi.
2. Acclimation medium: K_2_Cr_2_O_7_ and NaF were added to the enrichment medium and sterilized at 121°C for 20 min.
3. Solid medium: 2% agar was added to the enrichment medium and sterilized at 121°C for 20 min. (All chemicals and solvents were of analytical grade, and no further purification was required).

#### Test water quality

Considering the volatility and complexity of actual groundwater, the experimental water samples were configured to simulate the groundwater quality in the Fuxin mining area. The mass concentrations of SO_4_^2-^, Cr(VI), Cr(III) and F^-^ in the water samples (pH = 4.6) were 500 mg/L, 10 mg/L, 10 mg/L, and 5 mg/L, respectively, and thus, the total mass concentration of chromium in the solution was 20 mg/L.

#### Preparation of nano-ZrO_2_-polyacrylamide (ZP) hybrid materials

Two grams of zirconia was dissolved in 200 mL of 95% ethanol solution, and the reaction was carried out through hydrolysis and polycondensation. To obtain a colorless and transparent nanozirconia gelatin, 0.6 g of acrylamide monomer was added to the sol, heated in a water bath, and stirred evenly, and nitrogen was added for 30 minutes to remove dissolved oxygen. Then, 0.05 g each of sodium bisulfite and potassium persulfate (mass ratio 1:1) was added as initiators, and the mixed solution was heated to 25°C in a water bath and fully stirred to initiate polymerization. After 30 minutes of reaction, the reaction was stopped as the viscosity did not further change, and the solution was allowed to cool naturally to room temperature [29,30]. An organic-inorganic hybrid material with nanozirconia as the core and polyacrylamide as the shell was obtained.

### Test methods

#### Culture, acclimation, purification and isolation of the strain

An enrichment medium with sodium citrate as the carbon source was inoculated at 5% into the sludge. The enrichment culture was carried out in a biochemical incubator at 35°C. New medium was added every 7 days until a dominant strain was obtained. The strain was domesticated for chromium tolerance and fluorine tolerance so that it could grow in wastewater containing high concentrations of Cr(VI), Cr(Ⅲ) and F^-^. First, Cr(VI) and Cr(III) with a mass concentration of 5 mg/L and F^-^ with a mass concentration of 1 mg/L were added to the culture medium to make the bacteria gradually adapt to the growth environment. Then, Cr(VI), Cr(III) and F^-^ were added to the medium every 7 days by the gradient method until the strain adapted to an environment with mass concentrations of 100 mg/L Cr(VI), 100 mg/L Cr(III) and 20 mg/L F^-^.

Purification and isolation of the strains were performed by dilution coating and sandwich culture on dishes. The obtained colony morphologies and microscopic examination results were the same.

All operations were carried out in an anaerobic operating platform (Bactron Ⅱ, SHELLAB, USA).

#### Molecular biological identification of the strain

The morphology of the strain was observed by transmission electron microscopy (TEM), and the Gram staining results were observed by oil microscopy.

Colony genomic DNA was extracted by using a Maxwell 16 strain DNA purification kit. Polymerase chain reaction (PCR) amplification was carried out using a universal primer for bacterial 16S rDNA [31]. After purification and amplification of the product, the Beijing Liuhe Huada Gene Technology Service Co., Ltd. was commissioned to perform sequencing.

#### Immobilization of the strain

Sodium alginate (2.5%) was weighed into 300 mL of distilled water and allowed to fully swell. A certain amount of inorganic-organic hybrid material was added, mixed and dissolved, and the vessel was sealed and stored at room temperature for 8–12 h. Then, a 2.5% mass ratio of the pore-forming agent polyethylene glycol was added to the mixed solution, and a certain amount of bacterial liquid from the logarithmic phase after acclimatization and culture (the bacterial density of the bacterial liquid in the logarithmic phase was 3 × 10^8^ pcs/mL) was added. After full mixing, a syringe was used to drop the mixture into a 2% CaCl_2_ saturated boric acid solution at pH = 6, and stirring and crosslinking were performed at 100 r/min. After 4 h, the particles were removed and washed with 0.9% normal saline, and the surface moisture was absorbed [32,33]; this process was repeated 3 times. Before use, the pellets were placed in the enrichment medium and activated for 12 h.

#### Static single-factor experiment

In this study, a large number of preliminary experimental results show that temperature, dosage, and concentration are the main factors affecting the treatment effect, and the change in pH during the reaction process will also have a large degree of influence on the treatment effect. Therefore, the temperature, dosage, concentration and pH in the process are mainly considered in this study. To avoid repeated research on influencing factors, the influence of the temperature and dosage are discussed in the single-factor experiment section, and the influence of the concentration is considered in the dynamic experiment section. In addition, the pH of the reaction process was recorded during the entire study. In the experiment, a 200 mL water sample was treated at a solid-liquid ratio of 1:10 with pH = 4.6, a 35% volume fraction of Citrobacter in the logarithmic phase, and 300 mL of the ZP hybrid material. Using the single variable method, the effects of different dosages of Citrobacter (0%, 10%, 20%, 30%, 35%, 40%, and 45%) and different dosages of ZP hybrid material (0 mL, 100 mL, 200 mL, 300 mL, 400 mL, and 500 mL) at 35°C as well as the effect of different reaction temperatures (25°C, 30°C, 35°C, 40°C, and 45°C) on the removal efficiencies of SO_4_^2-^, Cr(VI), Cr(III) and F^-^ were measured every 5 h, and the removal rate was calculated. The removal rate of each pollutant could be calculated by the following formula:

$$\eta =\frac{C_{0}-C_{n}}{C_{0}}\times 100\%$$ (1)

where *η* is the removal rate of each pollutant, *C*_0_ (mg/L) is the initial concentration of each pollutant ion, and *C*_n_ (mg/L) is the concentration of each pollutant ion in the extract.

#### Dynamic experiment

**Fig 1:**
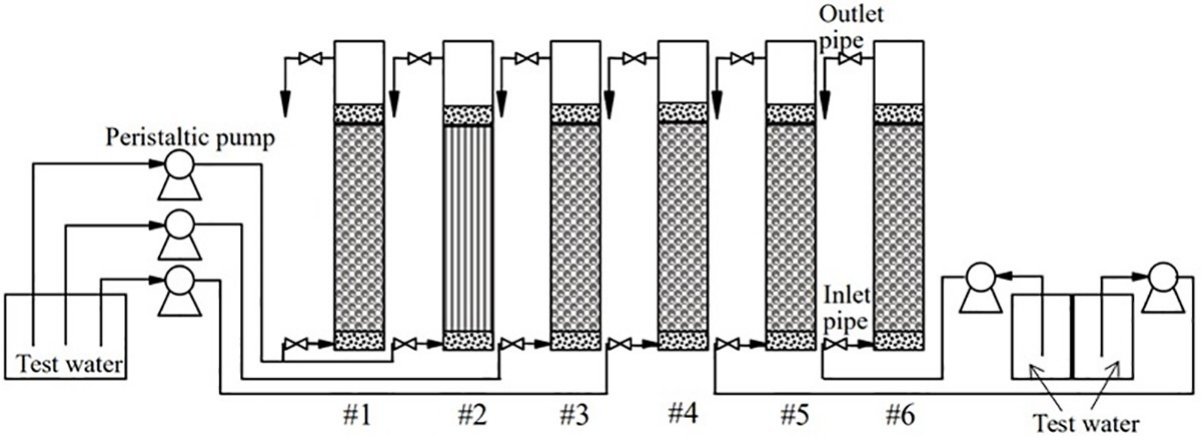
The dynamic test device.

Six groups of dynamic columns with an outer diameter of 50 mm, inner diameter of 45 mm and height of 30 cm were designed. The inlet area at the bottom was filled with 2 cm small white gravel, and the 20 cm above this layer was filled with a reaction layer. The 2 cm small white gravel was set above the reaction layer. The test device is shown in Fig 1. The test was carried out in a constant-temperature and constant-humidity laboratory, and the indoor temperature was kept at approximately 35 ± 1°C. The ZPC particles used in the test were made by using the best proportions determined in the static test, and the pH value of the influent water was 4.6 ± 0.1. The specific operating conditions of each dynamic column are shown in Table 1. The removal efficiency of each pollutant in the water was determined continuously.

**Table 1:** Operating conditions of the dynamic column experiment.

Dynamic column serial number	Filling material of reaction layer	Influent hydraulic load/m^3^·(m^2^·d)^-1^	concentration of SO_4_^2-^/mg/L	concentration of Cr(VI)/mg/L	concentration of Cr(III)/mg/L	concentration of total chromium/mg/L	concentration of F^-^/mg/L
#1	ZPC particles	3.0	500	10	10	20	5
#2	Citrobacter strain	3.0	500	10	10	20	5
#3	ZPC particles	1.5	500	10	10	20	5
#4	ZPC particles	4.5	500	10	10	20	5
#5	ZPC particles	3.0	500	50	10	60	5
#6	ZPC particles	3.0	500	10	10	20	10

Note: The filling material of the reaction layer of column #2 was Citrobacter bacterial solution, and the elastic filament was used as the filling material. The bacterial density was 3 × 10^8^ pcs/mL, which was the same as the concentration of Citrobacter bacterial solution used to make ZPC particles.

#### Microscopic characterization of ZPC particles

Dynamic light scattering (DLS) was used to measure the particle size of nano-ZrO_2_ gelatin. To prevent the agglomeration of particles, ultrasonic dispersion was used for 1 h to evenly disperse the nanoparticles in the sol. Scanning electron microscopy (JMS-7000F, JEOL, Japan) was used to analyze the surface morphology changes of the dried samples. A FTIR spectrometer (AVATAR 330, Thermo Electron Corporation, USA) was used to characterize the molecular structure, chemical bonds and functional group changes of the dried samples. X-ray diffraction (XRD-6100, SHIMADZU, Japan) was used for phase analysis of the dried samples. In order to verify the reducibility of the Citrobacter, ZPC particles were used to react with the composite water samples without adding Cr(Ⅲ). The reacted ZPC particles were dehydrated and subjected to XRD. The dehydration method was as follows. First, the prepared ZPC particles were added to 2.5% glutaraldehyde fixative and fixed for 1 hour. Then, ethanol at concentrations of 50%, 70%, 80%, 90%, 95%, and 100% was used for dehydration. Each dehydration time was 30 min, and each concentration was dehydrated twice. Next, the samples were soaked in 100% ethanol for 2 h and soaked in tert-butyl alcohol twice instead of ethanol. The samples were frozen and dried in vacuum for 24 h in a freeze dryer (FD-1a-50, Hefan, Shanghai, China) until the tert-butyl alcohol in the samples was completely volatilized.

#### Regeneration properties and reusability

To study the feasibility of regeneration and reuse of ZPC particles, a batch test was used to perform seven cycles of adsorption and elution. The ZPC particles were placed into 50 mL of eluent with 0.1 mol/L HCl, 0.2 mol/L ethanol and 2.5% thiourea and shaken at 35°C and 60 rpm for 12 h. Then, the mixture was placed into 100 mL of enriched medium containing a large amount of carbon source and regenerated at 35°C for 12 h to form an internal carbon source. After one cycle was completed, the ZPC particles were removed as described above and regenerated for the next cycle. The cycle test was repeated seven times, and the concentrations of SO_4_^2-^, Cr(VI), total chromium and F^-^ were detected.

### Water quality testing methods

The SO_4_^2-^ was determined by barium chromate spectrophotometry (V-1600PC, MAPADA, Shanghai). The Cr(VI) was determined by the dtphenylcarbohydrazide spectrophotometric method (UV-2550, SHIMADZU, Kyoto). The total chromium was determined by potassium permanganate oxidation-diphenylcarbazide spectrophotometry (UV-2550, SHIMADZU, Kyoto). The F^-^ was determined by the ion-selective electrode method (PHS-3C, LEICI, Shanghai), and the pH value was determined by a pH meter (PHS-3C, LEICI, Shanghai).

## Results and discussion

### Morphological characteristics of the strain

**Fig 2:**
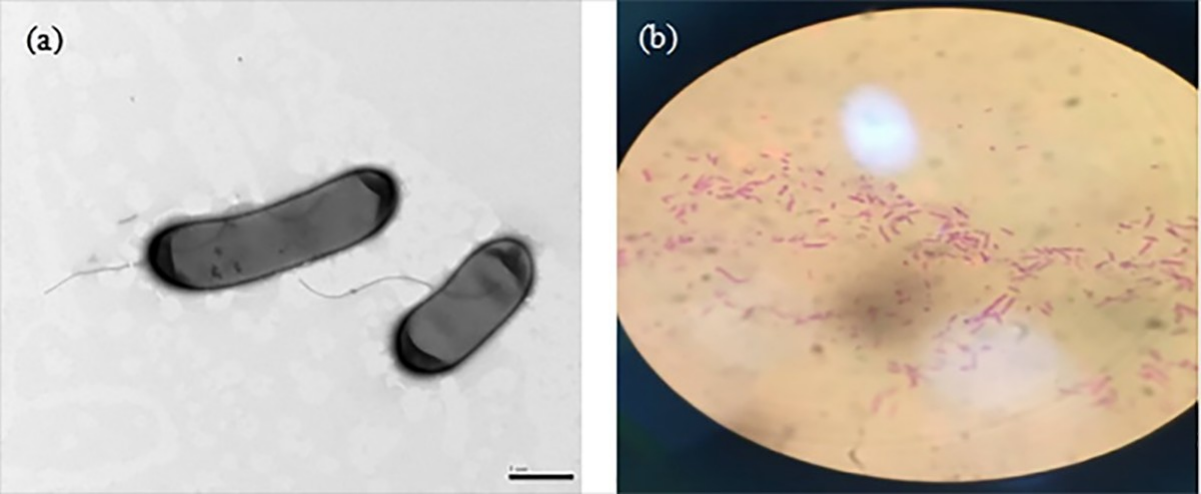
Morphological characteristics of the strain. **(a)** TEM image of the strain; **(b)** Gram-stained image of the strain.

The scanning electron microscopy (SEM) image in Fig 2(A) shows that the strain was rod-shaped, with a length of approximately 2–4 μm and a diameter of approximately 0.5–1 μm without spores or flagella. Fig 2(B) shows an image under 1600× oil lens magnification. The strain was stained red as a result of Lan’s staining, and thus, it was preliminarily judged that the bacterium was Gram negative. The obtained sequencing results were compared with the BLAST gene bank, and sequence homology analysis was performed. The results are shown in Table 2. From the table, this bacterium had the highest similarity with *Citrobacter amalonaticus* TB10, with a similarity of 99.93%, indicating that this strain was of the same nature as *Citrobacter amalonaticus* TB10.

**Table 2:** Sequence homology analysis.

Rank	Name	Strain	Similarity (%)
1	*Citrobacter amalonaticus*	TB10	99.93
2	*Citrobacter amalonaticus*	HAMBI 1296	99.86
3	*Citrobacter amalonaticus*	LMG 7873	99.78
4	Uncultured Citrobacter sp. clone	F2AUG.11	99.71
5	*Citrobacter farmeri*	CIP 104553	99.64
6	*Citrobacter farmeri*	17.7 KSS	99.57
7	Uncultured bacterium clone	KSR-CFL3	99.49
8	*Citrobacter amalonaticus*	OFF7	99.42
9	Citrobacter sp.	CF3-C	99.35
10	Citrobacter sp. enrichment culture clone	TB39-15	99.28

### Static test results and analysis

#### Effect of the Citrobacter dosage on the remediation of polluted groundwater by ZPC particles

**Fig 3:**
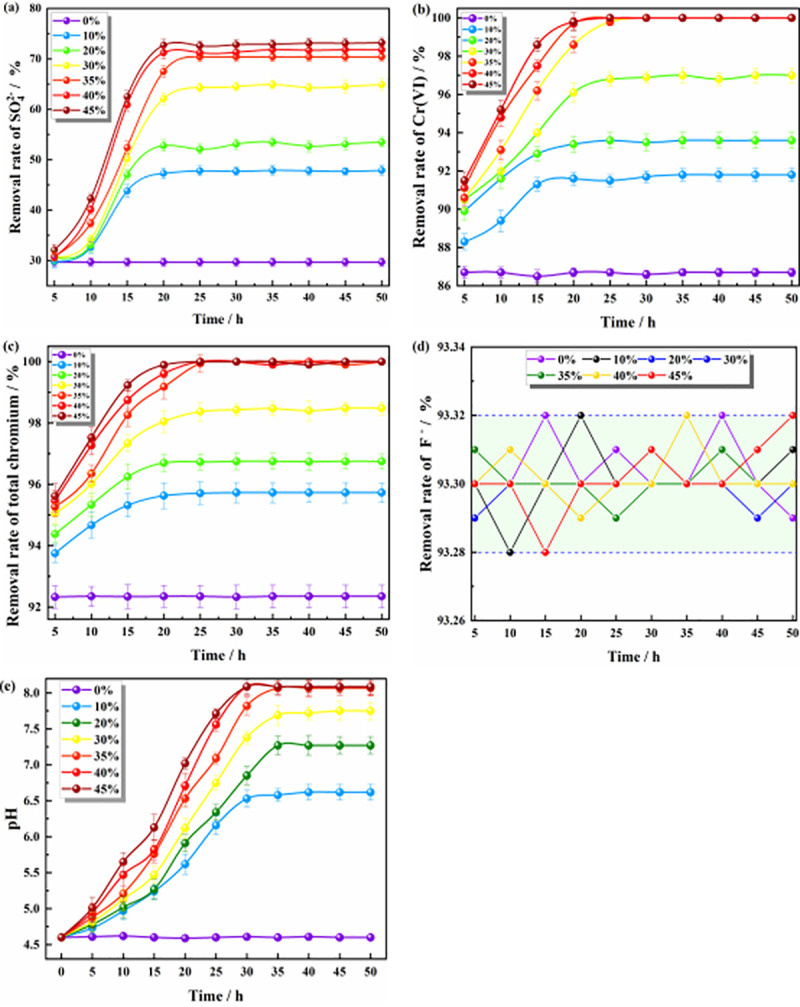
Effect of the dosage of Citrobacter on the removal of pollutants. **(a)** The effect on the removal of SO_4_^2-^; **(b)** The effect on the removal of Cr(VI); **(c)** The effect on the removal of total chromium; **(d)** The effect on the removal of F^-^; **(e)** The effect on pH value.

Fig 3 shows that with the progress of the reaction, the dosage of Citrobacter in the ZPC particles had a considerable impact on the SO_4_^2-^, Cr(VI), and total chromium contents and pH in the mine water but had no significant effect on the F^-^. Fig 3(A) shows the results for ZPC particles with different dosages of Citrobacter; when the dosage was small, the small amount of bacteria had poor adaptability to the water environment and a low survival rate [34], and the amount of SO_4_^2-^ reduced in the solution was also small. When the dosage of Citrobacter was 0%, the removal rate of SO_4_^2-^ was only 29.7%. When the dosage of Citrobacter was increased to 35%, the removal rate of SO_4_^2-^ reached 70.5%. Increasing the content of Citrobacter to 45% increased the removal rate of SO_4_^2-^ by only 2.7%. SO_4_^2-^ was mainly removed by the reduction effect of Citrobacter. Many studies have shown that Citrobacter has a significant removal effect on SO_4_^2-^ [35,36]. As seen from Fig 3(B), when the dosage of Citrobacter was 0%, the removal rate of Cr(VI) was more than 86%, and thus, it can be judged that the hybrid material can remove Cr(VI). Azeez et al. also found that nano-ZrO_2_ has a repairing effect on Cr(VI) [37]. At the beginning of the reaction, the amount of Cr(VI) reduced by Citrobacter was low, but when the dosage of Citrobacter was more than 35%, the final removal rate of Cr(VI) reached more than 100%. *Citrobacter freundii* separated from tanning wastewater by Vijayaraj et al. can reduce the concentration of Cr(VI) by 73% [38]. ZPC particles synthesized from Citrobacter and ZP have a good effect on Cr(VI) removal. Fig 3(C) shows that the dosage of Citrobacter had less of an effect on the removal of total chromium than on the removal of Cr(VI). Total chromium is composed of Cr(VI) and Cr(III). At the initial stage of the reaction, some Cr(III) was formed by the reduction of Cr(VI) by Citrobacter in the solution. At this time, Cr(III) in the solution was mainly removed by adsorption to the ZP material. The Zr-MMT nanomaterials prepared by Hei et al. have a good adsorption capacity for Cr(Ⅲ). Studies have shown that the main adsorption methods are ion exchange, electrostatic adsorption and surface adsorption [39]. Fig 3(D) shows that the dosage of Citrobacter had no significant effect on the removal of F^-^, and the removal rate of F^-^ was always maintained at approximately 93.3%. F^-^ was removed by ZP adsorption, and the dosage of Citrobacter did not affect the adsorption capacity of ZPC particles. Since the pH of groundwater in local mines is stable at approximately 4.6 year round, to better apply ZPC particles in practical engineering, the initial pH of this study was set to 4.6. Thathsara et al. prepared a novel tri-metal composite incorporating polyacrylamide (TCIP) and found that when the pH was 4.8, the removal effect of F^-^ was the most obvious [40]. Wang et al. found that when the pH was 4~5, the adsorption capacity of F^-^ on the ZrO_2_-MWCT adsorbent was the largest [41]. Therefore, the initial pH of this experiment is suitable for the repair of F^-^ by ZPC particles. Fig 3(E) shows that with the continuous progress of the reaction, the strain can increase the pH of the solution system during the process of growth and reproduction. When the dosage of Citrobacter was 0%~45%, the pH value of the solution was stable at 4.6, 6.62, 7.27, 7.75, 8.07, 8.09 and 8.09. Guo et al. also found this phenomenon in their research. *Citrobacter sp*. strain GW-M can increase the pH value of the solution system to 8.31 [42]. As more Citrobacter was added, H^+^ was consumed faster [43], and the pH of the solution gradually increased to an alkaline value. When the pH value is greater than 7.5, Cr(III) will be removed in the form of Cr(OH)_3_ precipitates in the solution [44]. When the dosage of Citrobacter was 35%, the pH was increased to 8.07. Therefore, Cr(III) can be removed by adsorption by hybrid materials and the formation of Cr(OH)_3_ precipitates. ZPC particles are composed of nano-ZrO_2_ hybrid material and Citrobacter. Nano-ZrO_2_ has excellent chemical inertness and a stable chemical structure in the pH range of 4~8, and it does not affect the adsorption effect of pollutants [45]. In conclusion, considering the effect of Citrobacter on the removal of pollutants and the improvement of the pH value, the optimal dosage of Citrobacter was 35%.

#### Effect of the hybrid material dosage on the remediation of polluted groundwater by ZPC particles

**Fig 4:**
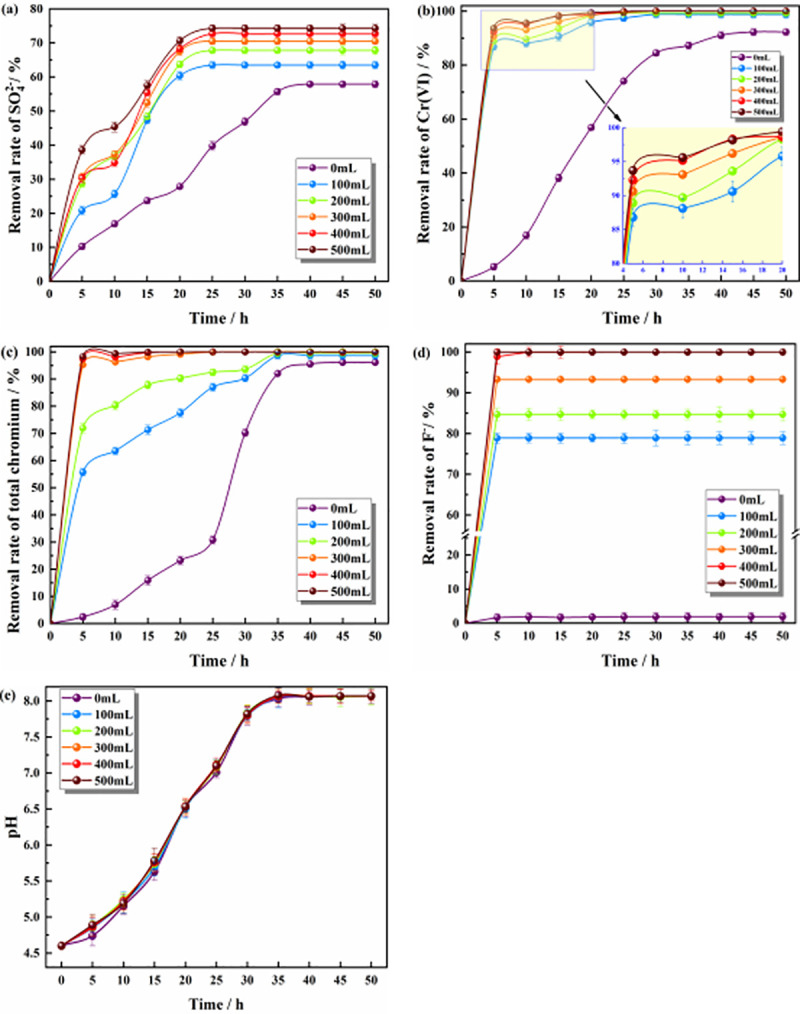
Effect of different dosages of hybrid materials on pollutants. **(a)** The effect on the removal of SO_4_^2-^; **(b)** The effect on the removal of Cr(VI); **(c)** The effect on the removal of total chromium; **(d)** The effect on the removal of F^-^; **(e)** The effect on the pH value.

Fig 4 shows that with the progress of the reaction, the removal rates of SO_4_^2-^, Cr(VI), total chromium, and F^-^ and the pH value in solutions with different dosages of hybrid materials gradually increased and finally tended to be stable. Fig 4(A) shows that when the dosages of hybrid material were 0 mL and 500 mL, the final removal rates of SO_4_^2-^ were 57.9% and 74.3%, respectively. Chen found that adding polyacrylamide would increase the removal rate of SO_4_^2-^ in mine wastewater [46]. Therefore, with an increasing dosage of hybrid material, the removal rate of sulfate increased less. The reduction of SO_4_^2-^ by Citrobacter was the main factor, and adsorption by the ZP material was the secondary factor. As shown in Fig 4(B), when Citrobacter was not adapted to the water environment 5 h before the reaction, the addition of ZP particles had a better removal effect on Cr(VI), which indicated that the ZP materials had an adsorption effect on Cr(VI). The ZrO_2_ prepared by Wu et al. has a maximum adsorption capacity of 25.27 mg/g for Cr(VI) [47]. The ZP material had a strong adsorption capacity for Cr(VI) and could effectively reduce the concentration of Cr(VI) in solution, reduce the toxicity of Cr(VI) to Citrobacter, and provide a good environment for the growth of Citrobacter. As the reaction proceeded, Cr(VI) was also reduced to Cr(III) by Citrobacter. Fig 4(B) shows that the dosage of ZP material had little effect on the final removal rate of Cr(VI). Fig 4(C) shows that the dosage of ZP material had little effect on the final removal rate of total chromium because some of the Cr(VI) in the solution was adsorbed by ZP, and some of it was reduced to Cr(III) by Citrobacter. Some Cr(III) could be adsorbed by ZP, and some could be removed by the precipitation of Cr(OH)_3_. Therefore, the effect of the ZP dosage on the total chromium was small. The nano-ZrO_2_ composite prepared by Mahmoud et al. also has the ability to simultaneously adsorb Cr(VI) and Cr(III) [48]. Fig 4(D) shows that the addition of the ZP material had a great influence on F^-^. When the dosage of ZP was 0 mL, the removal rate of F^-^ was less than 2%, with no significant effect. When the dosage of ZP was 300 mL, the removal rate of F^-^ was 93.3%, and when the dosage increased to 400 mL, the removal rate of F^-^ was 100%. The nano-ZrO_2_ composite material developed by Mohan et al. also showed a good adsorption capacity for F^-^, with a maximum adsorption capacity of up to 45 mg/g [49]. Fig 4(E) shows that the dosage of ZPC had no significant effect on the pH value of the solution, indicating that the hybrid material had no adsorption effect on H^+^ and that the pH value of the solution was increased by Citrobacter. When the dosage of the ZP material was 300 mL, the removal rates of SO_4_^2-^, Cr(VI), total chromium and F^-^ were 70.5%, 100%, 100% and 93.3%, respectively. On this basis, increasing the dosage of the ZP material could slightly improve the removal rates of pollutants. Considering the economic cost and other factors, the optimal dosage of the ZP material was 300 mL.

#### Effect of the reaction temperature on the remediation of polluted groundwater

**Fig 5:**
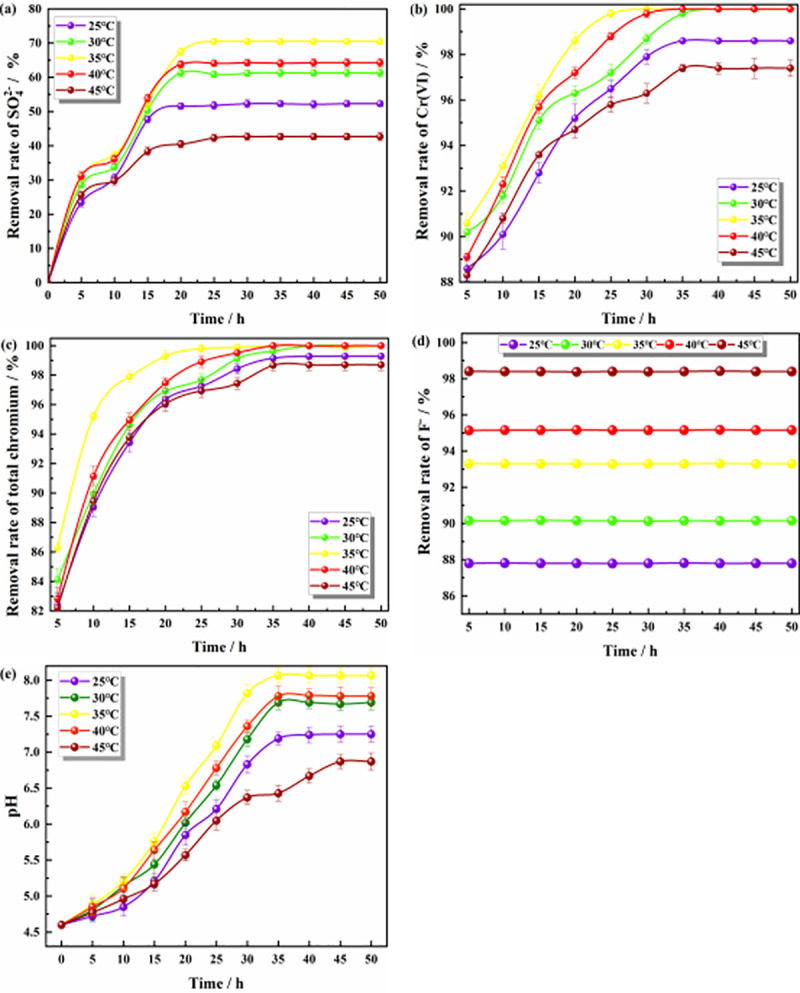
Effect of the reaction temperature on pollutants. **(a)** The effect on the removal of SO_4_^2-^; **(b)** The effect on the removal of Cr(VI); **(c)** The effect on the removal of total chromium; **(d)** The effect on the removal of F^-^; **(e)** The effect on pH value.

As shown in Fig 5, with the progress of the reaction, the removal rates of SO_4_^2-^, Cr(VI), total chromium, and F^-^ and the pH value in the solution at different temperatures gradually increased and finally tended to be stable; among the investigated parameters, the temperature had a greater impact on the SO_4_^2-^, F^-^ and pH values. Fig 5(A) shows that the order of the SO_4_^2-^ removal rate from large to small at the five temperatures was 35°C > 40°C > 30°C > 20°C > 45°C. The best reaction temperature was 35°C, at which the Citrobacter activity was the strongest [50], and the maximum SO_4_^2-^ removal rate was 70.5%. This is because Citrobacter is a mesophilic bacterium [51], and a temperature of approximately 35°C is the optimal growth temperature for the strain [10]. At this temperature, Citrobacter has the strongest biological activity and the most vigorous growth and metabolic ability. Too high or too low a temperature is not conducive to the growth and reproduction of Citrobacter. When the temperature is too low, the activities of various enzymes in cells are reduced, fewer metabolic products are produced, and the removal rate of pollutants is low. When the temperature is too high, the proteins and nucleic acids of bacterial cells will denature and inactivate, thereby affecting the pollutant removal effect of the bacteria [52,53]. Zhou et al. also found that the strain had the best biofilm formation effect and the best removal effect on pollutants at the optimum temperature [54]. Ojha also showed similar results in his study. When the temperature was 37°C, the strain had the highest amylase production [55]. Fig 5(B) shows the optimum temperature for ZPC to adsorb Cr(VI) at approximately 35°C. At this temperature, the ability to reduce Cr(VI) and adsorb Cr(VI) on ZP was the strongest. Research by Gusain et al. showed that 32.8°C is the best temperature for nano-ZrO_2_ to adsorb Cr [56]. Teimouri et al. found that 35°C is the best reaction temperature for nano-ZrO_2_ and nano-ZrO_2_ composites to adsorb nitrate, and the adsorbent has the best adsorption effect on pollutants at this temperature [57]. Fig 5(C) shows that the temperature had little effect on the final removal rate of total chromium. Although temperature will affect the reduction of Cr(VI) and the increase in the pH value by Citrobacter, Cr(VI) and Cr(III) were removed by the combined action of ZP adsorption and Citrobacter. The study showed that F^-^ relied on the adsorption of ZP material for removal, and Citrobacter had no effect on F^-^. Fig 5(D) shows that as the reaction temperature increased, the removal rate of F^-^ also increased. Since the adsorption of F^-^ by ZP particles is an endothermic reaction, the higher the temperature is, the better the adsorption effect is [58]. Therefore, temperature has a great influence on the removal of F^-^. Wang et al. used ZrO_2_ composite materials as adsorbents. In their research, they found that the adsorption capacity of the adsorbent for F^-^ increased with increasing reaction temperature [59]. Fig 5(E) demonstrates that the effect of the pH was the best when the temperature was 35°C, and the pH of the solution system could be increased from 4.6 to 8.07. In addition, 35°C was the optimal growth temperature of bacteria [34], and the growth and metabolism of strains was the strongest at this temperature; too high or too low of a temperature is not conducive to alkali production by Citrobacter [43]. In the study, Li et al. found that the strain *Shewanella putrefaciens* CN32 can increase the pH value of the system to the highest value under the optimum temperature [60]. Therefore, the optimal reaction temperature was determined to be 35°C.

### Dynamic test results and analysis

**Fig 6:**
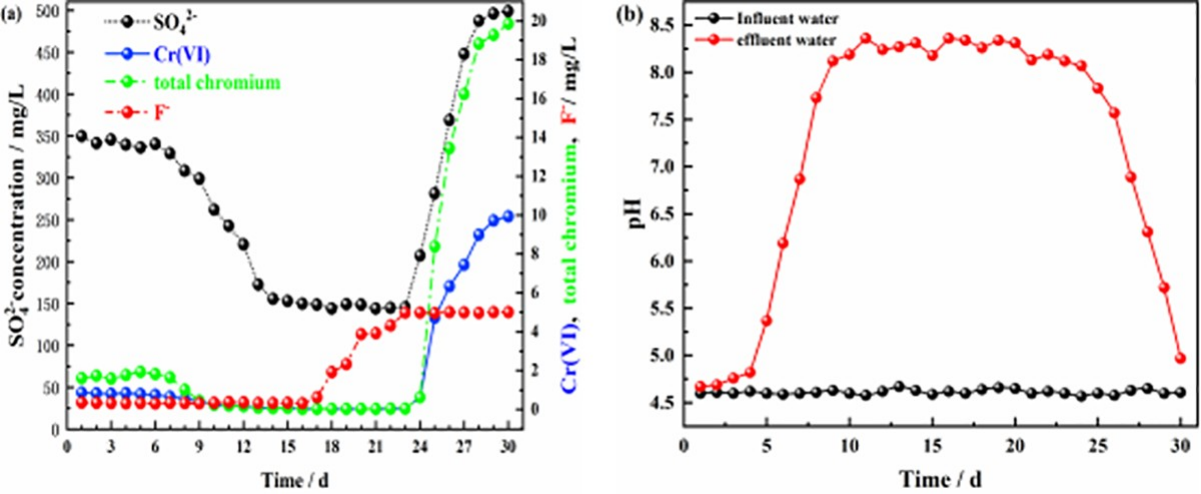
Effluent of dynamic column #1. **(a)** Effluent concentration of each pollutant ion; **(b)** pH values of influent and effluent water.

**Fig 7:**
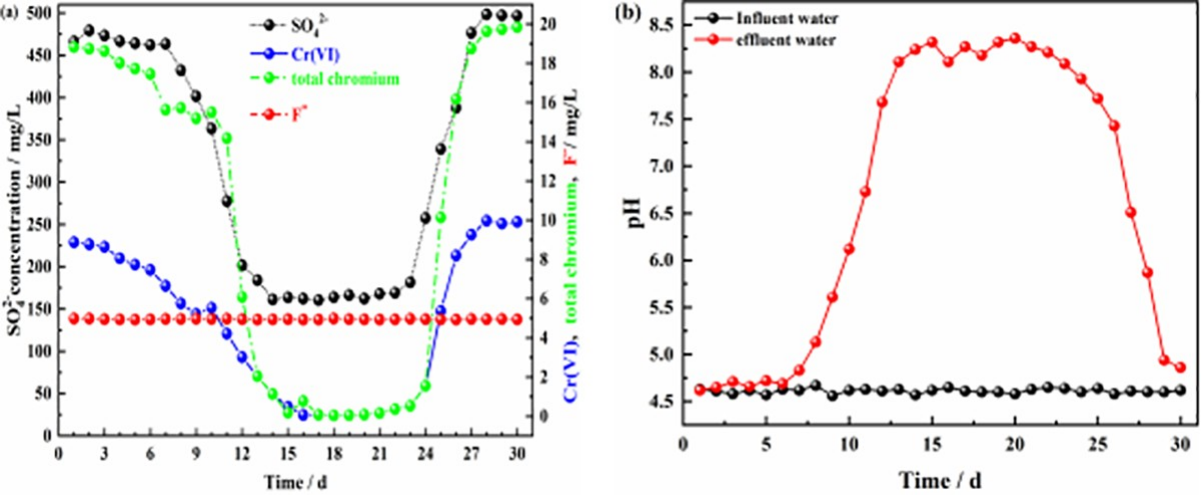
Effluent of dynamic column #2. **(a)** Effluent concentration of each pollutant ion; **(b)** pH values of influent and effluent water.

**Fig 8:**
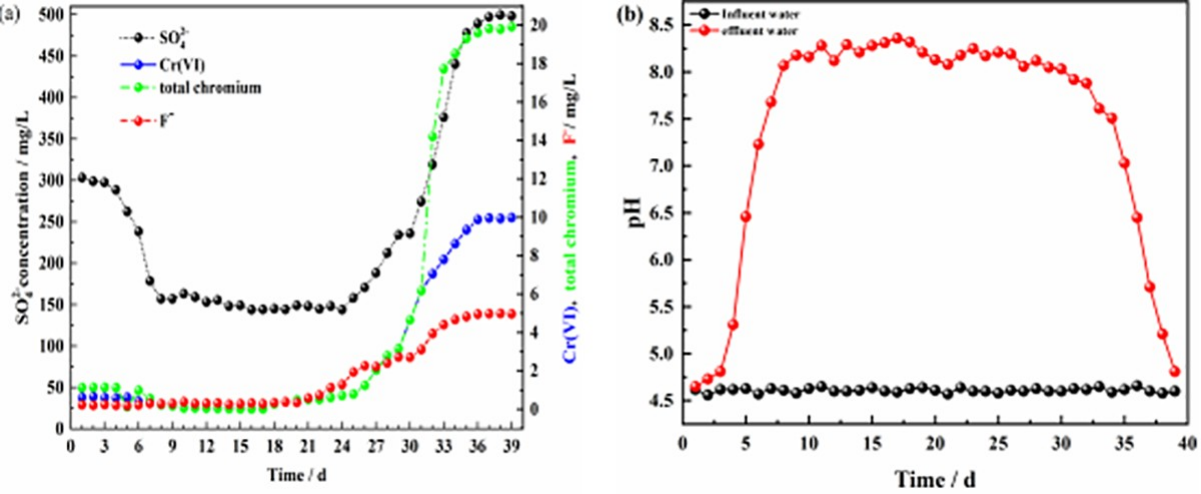
Effluent of dynamic column #3. **(a)** Effluent concentration of each pollutant ion; **(b)** pH values of influent and effluent water.

**Fig 9:**
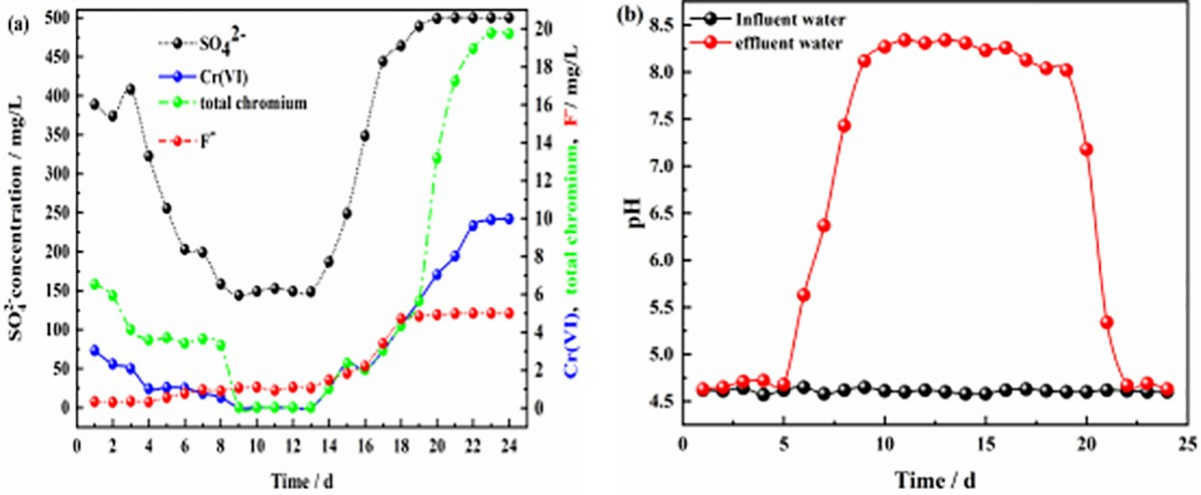
Effluent of dynamic column #4. **(a)** Effluent concentration of each pollutant ion; **(b)** pH values of influent and effluent water.

**Fig 10:**
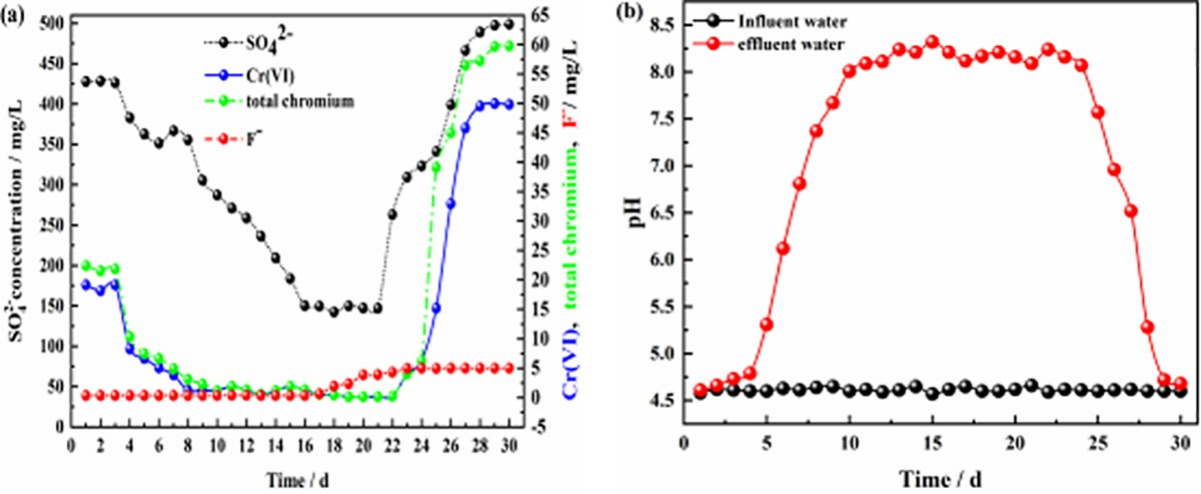
Effluent of dynamic column #5. **(a)** Effluent concentration of each pollutant ion; **(b)** pH values of influent and effluent water.

**Fig 11:**
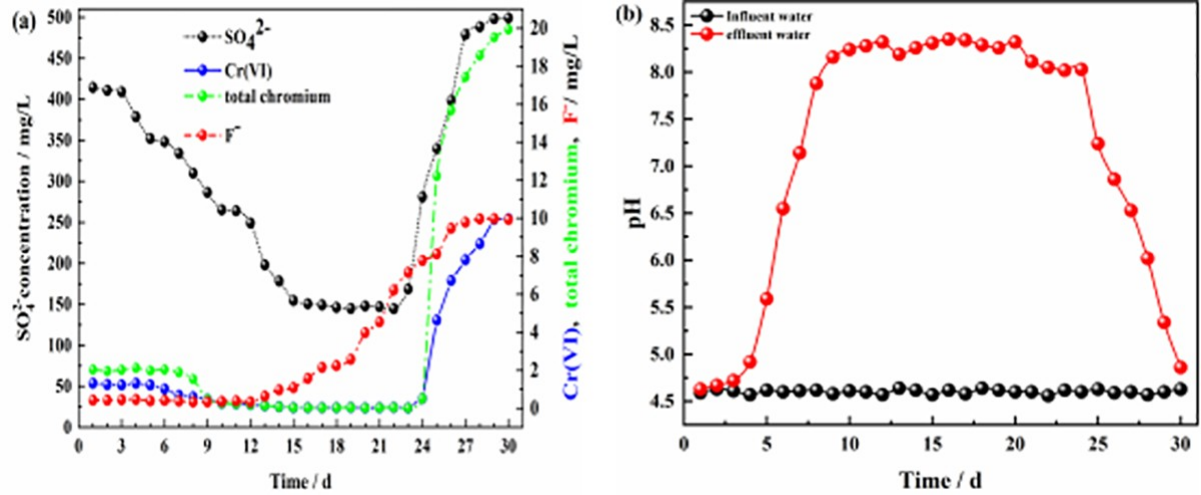
Effluent of dynamic column #6. **(a)** Effluent concentration of each pollutant ion; **(b)** pH values of influent and effluent water.

A schematic of the dynamic experiment is shown in Fig 1, and the results for the effluents from the six columns are shown in Figs 6–11.

Figs 6(A) and 7(A) show the effluent characteristics of dynamic columns #1 and #2. The removal effect of the ZPC particle reaction layer on SO_4_^2-^, Cr(VI), total chromium, and F^-^ was better than the removal effect of film-coated Citrobacter. The removal of SO_4_^2-^, Cr(VI), total chromium and F^-^ in solution by the ZPC granular reaction layer occurred under the dual action of Citrobacter and the ZP material, while the removal of F^-^ depended on adsorption by the ZP material. The maximum removal rates of SO_4_^2-^, Cr(VI), total chromium and F^-^ by dynamic column #1 were 71.20%, 99.7%, 99.85% and 94.00%, respectively. The maximum removal rates of SO_4_^2-^, Cr(VI) and total chromium by dynamic column #2 were 67.76%, 99.50% and 99.75%, respectively, and there was no removal effect on F^-^. In the early stage of the reaction, Citrobacter had not adapted to the water environment, could reduce only a small amount of Cr(VI) to Cr(III), and could not remove Cr(III), and thus, the total chromium concentration in the solution was relatively large. However, as the reaction progressed, Citrobacter gradually adapted to the water environment; the bacteria could reduce a large amount of Cr(VI), and as the pH value of the solution rose, Cr(III) formed Cr(OH)_3_ precipitates in the alkaline environment. The concentration of total chromium in the solution was greatly reduced.

As the reaction proceeded, the pH value of column #1 (Fig 6(B)) was stable at approximately 4.7 on days 1–4, which may be because Citrobacter had not adapted to the water sample and was in the growth retardation stage. After 5–8 days, the pH value of the solution gradually increased from 4.6 to 7.73; at this time, the bacteria entered the logarithmic growth period, and the pH value of the solution increased significantly, which may be related to the large amount of H_2_S gas produced by the bacteria during SO_4_^2-^ reduction and the large consumption of H^+^ in the solution. From days 9–24, the bacteria entered the stable period, and the pH value fluctuated slightly from 8.0–8.36. From days 25–30, most of the Citrobacter declined, and the pH value decreased from 7.83 to 4.97. The results showed that ZPC particles can improve the pH value of acidic wastewater. The pH value of column #2 (Fig 7(B)) increased gradually from 4.6 to 7.68 from days 1–12, and the Citrobacter in column #2 had a longer adaptation period than those in column #1. The pH value fluctuated slightly from 8.09 to 8.36 from days 13–23 and decreased from 7.93 to 4.86 from days 24–30. Comparing the pH values of the two columns, it can be seen that there was no significant difference in the maximum improvement in pH value, indicating that the pH value in the solution mainly depended on the effect of Citrobacter, while the ZP material had no effect on the improvement in pH value. The optimal pH range of column #1 was wider than that of column #2, which indicated that the ZP material could prolong the stable period of bacteria.

Figs 6(A), 8(A) and 9(A) show the effluents of dynamic columns #1, #3, and #4. Different influent hydraulic loads did not affect the maximum removal rates of SO_4_^2-^, Cr(VI), total chromium, and F^-^ but delayed or advanced the breakthrough time. Fig 6(A) shows that when the influent hydraulic load was 3.0 m^3^/(m^2^·d), the removal rate of F^-^ was maintained at the maximum level from days 1–16, and the removal rates of SO_4_^2-^, Cr(VI) and total chromium were maintained at the maximum level from days 14–23. Fig 8(A) shows that when the influent hydraulic load was 1.5 m^3^/(m^2^·d), the removal rate of F^-^ was maintained at the maximum level from days 1~20, and the removal rates of SO_4_^2-^, Cr(VI) and total chromium were maintained at the maximum levels from days 8–25. Fig 9(A) shows that when the hydraulic load was 4.5 m^3^/(m^2^·d), the removal rate of F^-^ was maintained at the maximum value only for the first 4 days, and the removal rates of SO_4_^2-^, Cr(VI) and total chromium were maintained at the maximum levels only from days 9–13. According to the relative ratios of Figs 6(B), 8(B) and 9(B), with the increase in influent hydraulic load, the time for maintaining the optimal pH range was shortened. Therefore, with increasing influent hydraulic load, the effective removal time for each pollutant by ZPC particles was significantly shortened. This is because under the same conditions of other influencing factors, the greater the hydraulic load of the influent is, the greater the mass transfer driving force on the reaction layer, which will shorten the contact time between the pollutants in the water sample and the reaction layer; this is not conducive to the diffusion and adsorption of ions. Thus, the mass transfer efficiency of the adsorbate is negatively affected, and pollutants may flow out of the dynamic column without full contact and reaction with the reaction layer. The influent hydraulic load should not be too small; too small a hydraulic load will prolong the contact time between the pollutants and reaction layer, allow only a small amount of water to be treated, easily produce liquid-phase longitudinal backmixing in the column bed [61], and reduce the effective utilization rate of ZP particles in the reaction layer. Therefore, the optimal influent hydraulic load was 3.0 m^3^/(m^2^·d).

When the concentration of Cr(VI) increased to 50 mg/L, the maximum removal rate of Cr(VI) by ZPC particles was still maintained at 99.7% according to the results for column #5 (Fig 10(A)). However, due to the low activity of Citrobacter during the initial 1–3 days, the removal rate of Cr(VI) in the effluent of dynamic column #5 was approximately only 62%, and the removal rate of total chromium was approximately 64%, which was significantly lower than that of dynamic column #1. In the initial stage of the reaction, Cr(VI) in the solution was mainly removed by the ZP material, but the adsorption of the ZP material for Cr(VI) in the solution was poor. As the reaction proceeded, Citrobacter gradually recovered its activity, and its reduction effect maintained the Cr(VI) concentration in solution at a better level. When the concentration of Cr(VI) increased, at the initial stage of the reaction, ZPC particles affected the removal rates of SO_4_^2-^, Cr(VI) and total chromium, inhibited the time to maintain the optimal range of pH values (Fig 10(A) and 10(B)), and had no effect on the removal of F^-^. Fig 11(A) shows that when the F^-^ concentration was increased to 10 mg/L, dynamic column #6 was in the initial stage of the reaction from days 1–3; in contrast to the results for dynamic column #1, the removal rate of F^-^ increased from 93% to approximately 96%, the removal rate of Cr(VI) decreased from 92% to approximately 87%, the removal rate of total chromium decreased from 92% to approximately 90%, the removal rate of SO_4_^2-^ decreased from 31% to approximately 18%, and the removal effect of the pH value (Fig 11(B)) was basically unchanged. The results showed that when the concentration of F^-^ in the solution increased, the adsorption of Cr(VI) and SO_4_^2-^ by the ZPC particles was affected in the initial stage of the reaction, and the effect on total chromium was small. Since Cr(VI) is an anion group in water, it has a competitive adsorption relationship with SO_4_^2-^ and F^-^ anions. The selectivity of ZPC particles to anions in the solution was different, and the adsorption selectivity was in the order F^-^ > Cr(VI) > SO_4_^2-^.

### Microscopic characterization analysis of ZPC particles

**Fig 12:**
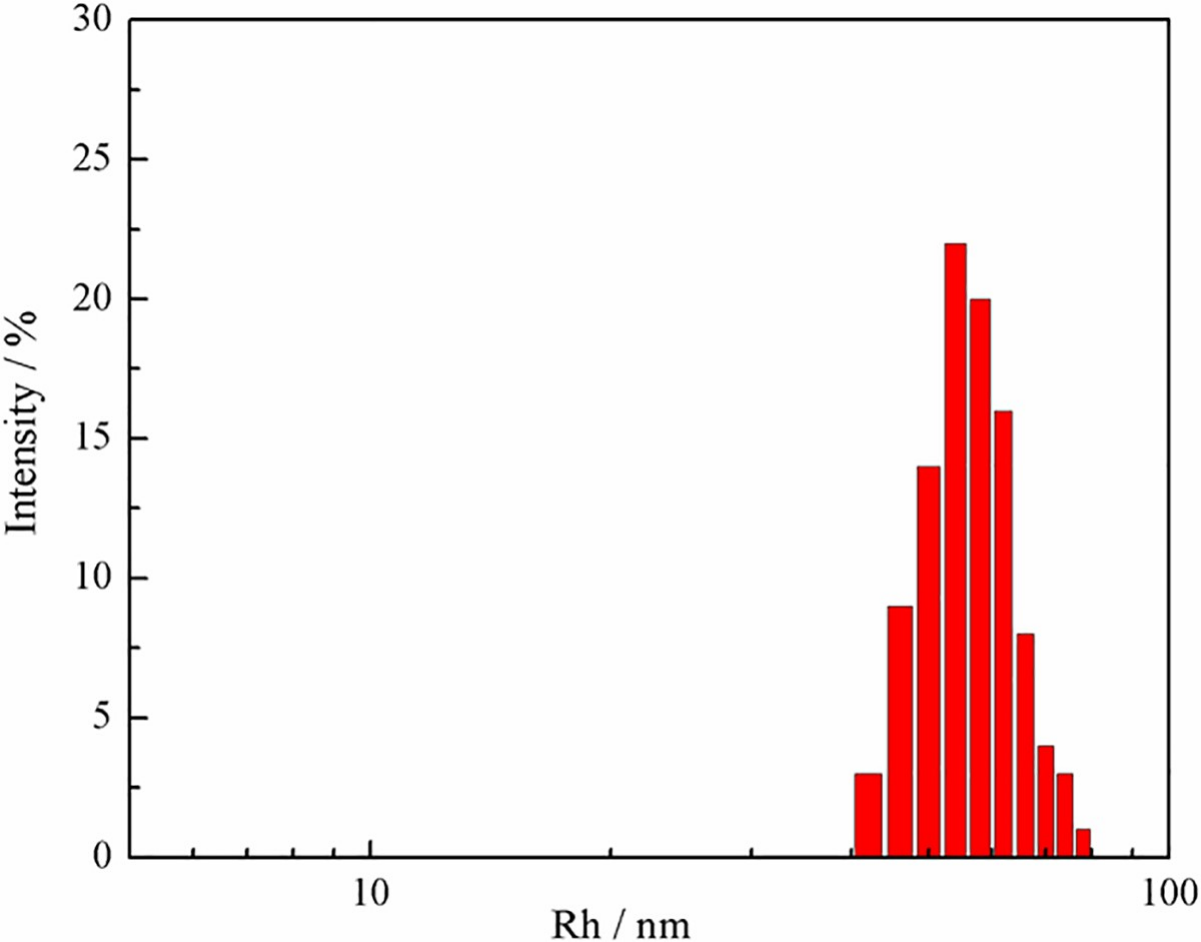
DLS results of nano-ZrO_2_ gelatin.

The hydrodynamic particle size and size distribution of nano-ZrO_2_ gelatin were determined by DLS, as shown in Fig 12. The results showed that the average particle size of the nanozirconia gelatin solution was 30–60 nm, which belonged to the nano range.

**Fig 13:**
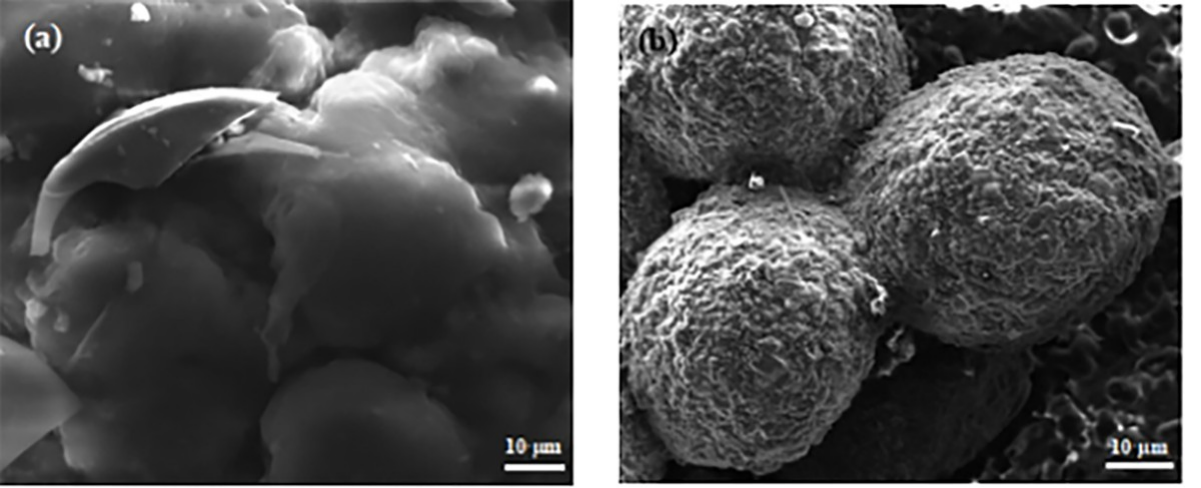
SEM images of ZPC particles. **(a)** Image before the adsorption reaction; **(b)** Image after the adsorption reaction.

**Fig 14:**
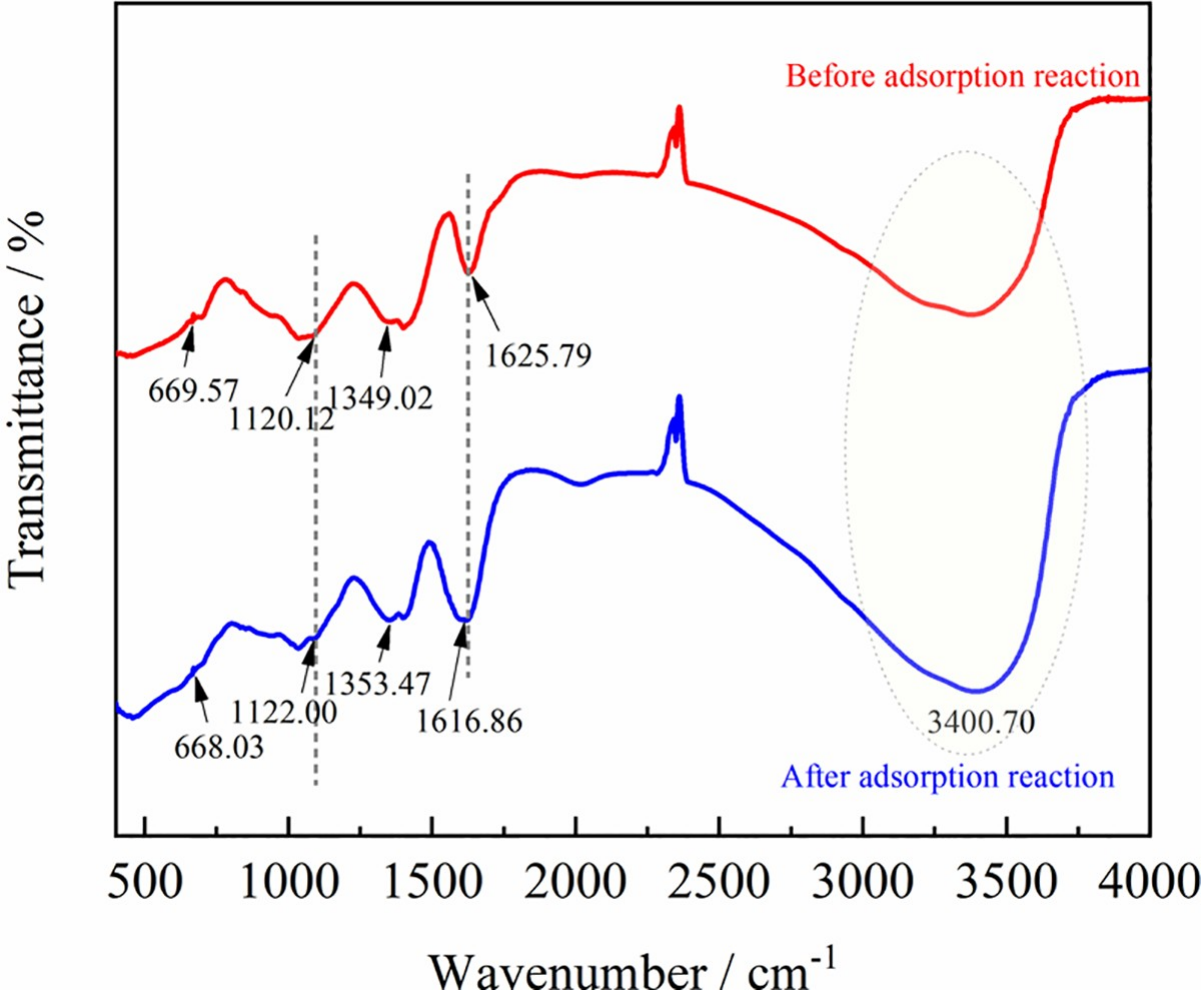
FTIR spectra of ZPC particles before and after the adsorption reaction.

**Fig 15:**
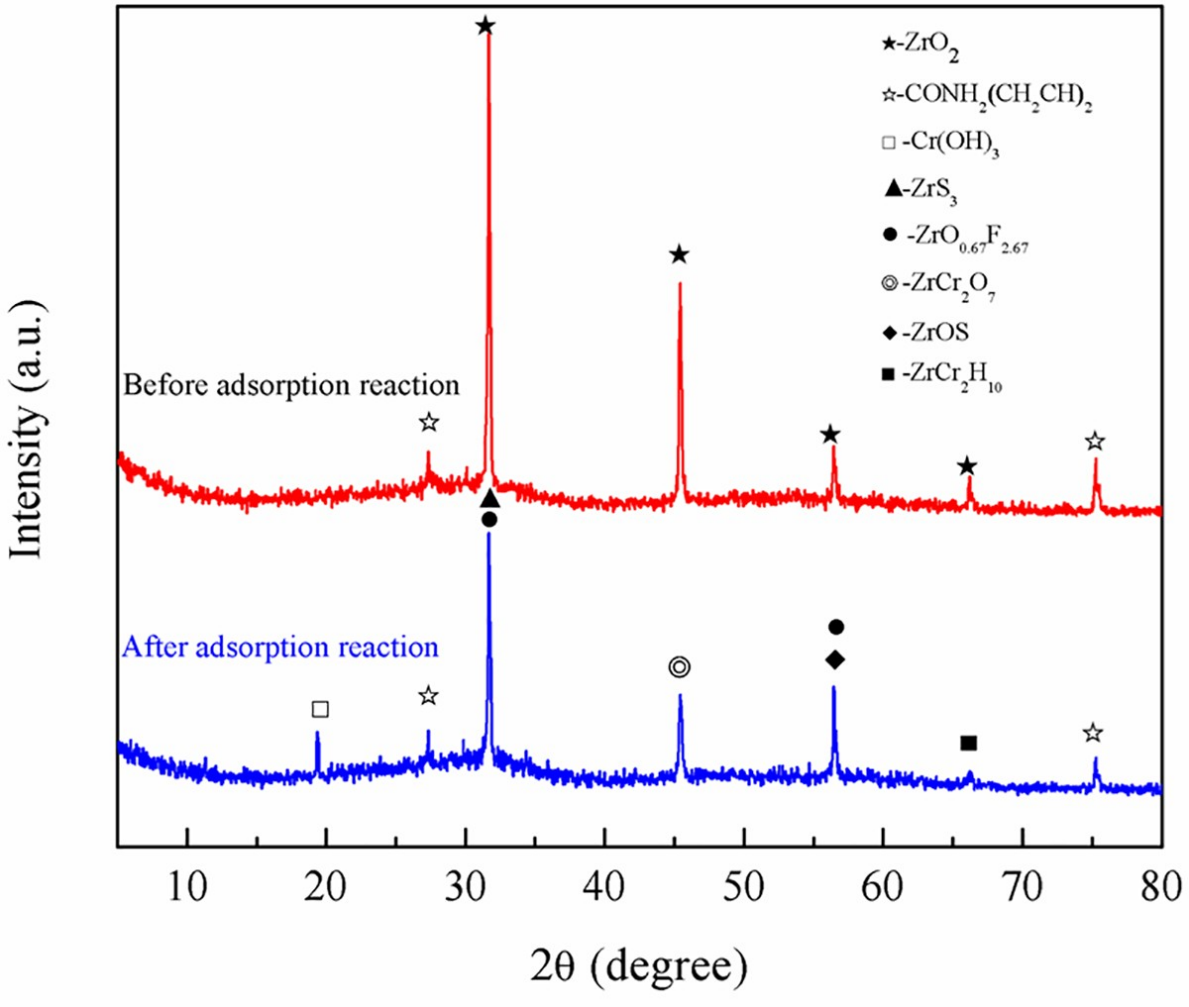
XRD patterns of ZPC particles before and after the adsorption reaction.

To further investigate the removal mechanism of pollutants by ZPC particles, Figs 13–15 show a comparative analysis before and after the pollutants are treated.

The comparative analysis before and after pollutant treatment is shown in Fig 13. SEM was used to observe the apparent structure of the material at 500 times magnification. Fig 13(A) indicates the apparent structure of ZPC particles before the reaction. ZPC particles have a porous structure, obvious pores, large pores, good dispersion and a large specific surface area. This structure makes ZPC particles suitable for the growth and attachment of microorganisms, with good microbial capacity, which can provide more space and higher mass transfer capacity for microbial growth. The apparent structure of ZPC particles after the adsorption reaction can be seen in Fig 13(B). It can be observed that the microspheres of ZPC particles after treatment of polluted water with ZPC particles became less obvious, the surface became rough and expanded, and a large number of folds appeared. The wrinkled surface structure can increase the contact area between ZPC particles and pollutants in the solution system and can quickly and efficiently remove pollutants in wastewater.

Fig 14 shows that before the adsorption reaction, the hybrid system had an obvious absorption peak at 669.57 cm^-1^, which corresponded to the characteristic absorption peak of Zr-O-Zr [62]. This indicates that there were characteristic absorption peaks of inorganic substance compounds in the hybrid materials. The peak at 1120.12 cm^-1^ was the -C-C stretching vibration, the peak at 1349.02 cm^-1^ corresponded to the -C-N stretching vibration, the peak at 1625.79 cm^-1^ was the -C = O stretching vibration of the amide group, and the broad peak at 3200–3700 cm^-1^ corresponded to the stretching vibration of amino -NH_2_ [63,64]. The characteristic peak of Zr-O-Zr, and the stretching vibration of -C-C, -C-N, -C = O, -NH_2_ shifted marginally after the reaction. These changes indicated that there may be reactions between the pollutants and the functional groups on the surface of the ZPC particles.

Fig 15 demonstrates that before the reaction, the main components contained in the ZPC particles are ZrO_2_ and the organic substance CONH_2_(CH_2_CH)_2_. After the reaction, the diffraction peak values decreased at angles of 30.08°, 45.40°, 65.20° and 75.28°, which indicated that the crystal structures of ZrO_2_ and CONH_2_(CH_2_CH)_2_ were destroyed. After ZPC particles were treated with compound polluted water, new substances such as Cr(OH)_3_, ZrS_3_, ZrO_0.67_F_2.67_, ZrCr_2_O_7_, ZrOS and ZrCr_2_H_10_ appeared on the basis of the original substances. Therefore, S mainly existed in the form of S^2-^ after reaction, while Cr mainly existed in the forms of Cr(VI) and Cr(III), which indicated that Citrobacter can reduce SO_4_^2-^ to S^2-^, Cr(VI) to Cr(III). Cr(III) and S^2-^ in solution reacted with other substances in particles and finally existed in the forms of Cr(OH)_3_, ZrCr_2_H_10_, ZrS_3_ and ZrOS, respectively. ZPC particles could directly adsorb Cr(Ⅵ), and the adsorbed Cr(Ⅵ) eventually existed in the form of ZrCr_2_O_7_ in ZPC. Since Citrobacter had no effect on the removal of F^-^ in water, F^-^ was finally adsorbed and removed by the hybrid material in the form of ZrO_0.67_F_2.67_. These results further clarify that ZPC particles have obvious removal effects on SO_4_^2-^, Cr(VI), Cr(III), F^-^.

### Regeneration properties and reusability

**Fig 16:**
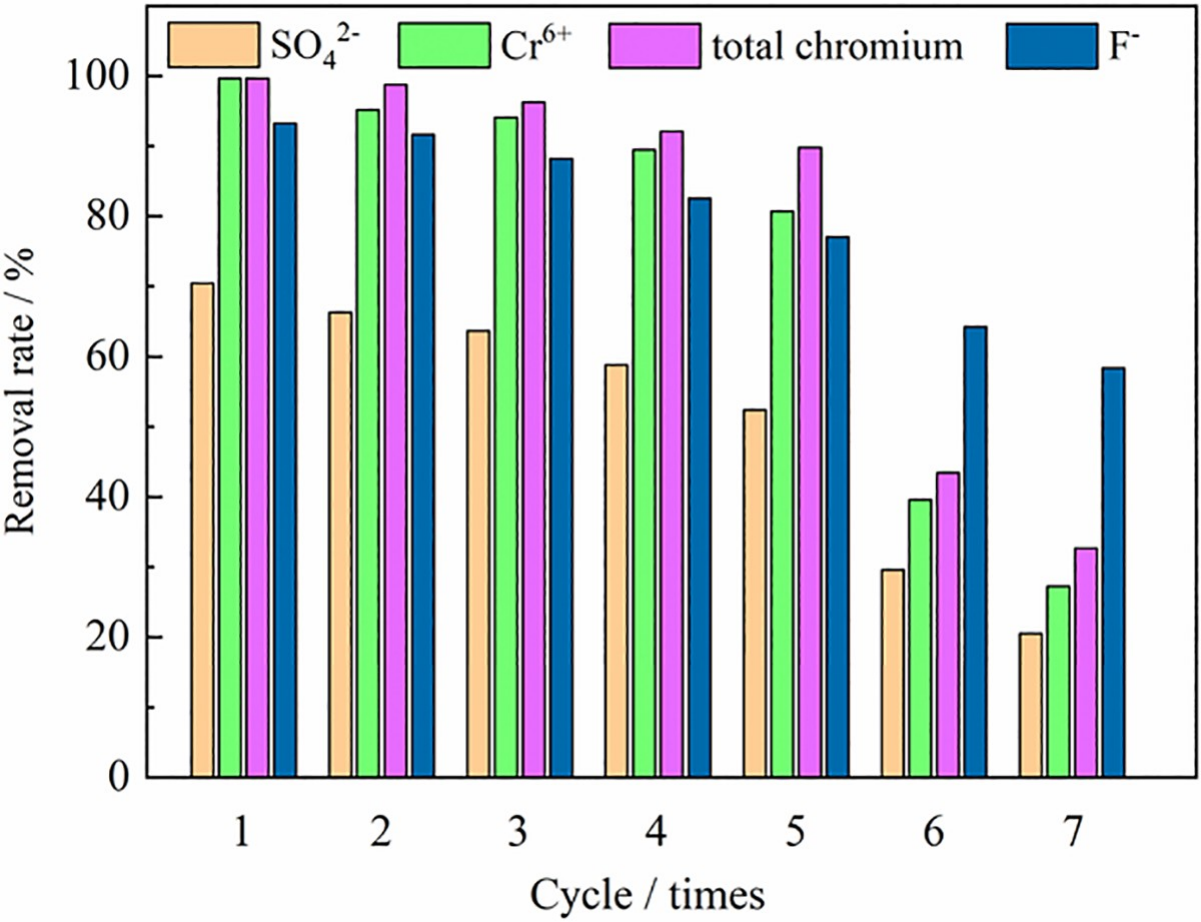
Stability and reusability of ZPC particles for up to 7 cycles.

Shukla et al. [65] believed that the recyclability of the adsorbent was critical to the long-term applicability of pollutant removal. This can be evaluated by comparing the removal performance of regenerated ZPC with the original ZPC. Using 0.1 mol/L HCl, 0.2 mol/L ethanol, and 2.5% thiourea as the eluents [66], the ZPC particles were tested for 7 cycles, and the results are shown in Fig 16. As the number of cycles increased, it was observed that the removal rate of each ion by the ZPC particles gradually decreased. Compared with the first cycle, after the fifth regeneration cycle, it was observed that the removal rates of SO_4_^2-^, Cr(VI), total chromium and F^-^ decreased from 70.5%, 99.7%, 99.7%, and 93.3% to 52.4%, 83.7%, 89.9% and 77.1%, respectively. The main reason for the decrease in adsorption capacity was that there was chemical adsorption in the adsorption process of the adsorbent for pollutants, and the adsorbed pollutants did not reach complete elution. The elution of the ZPC particles was insufficient during the regeneration cycle, resulting in ions remaining in the adsorption sites and reducing the adsorption performance of the ions, resulting in a decrease in the adsorption capacity [41]. From the sixth cycle, the pollutant removal rate decreased rapidly. The HCl eluent might destroy the cell structure of Citrobacter. Bajpai et al. also obtained similar conclusions when studying the stability of graphene aerogels for potential applications [67]. We observed that before 7 cycles, the ZPC particles could still maintain their shape without weight loss, which was very beneficial for recycling. These results showed that ZPC particles had good stability and reusability for potential applications.

## Conclusion

Homemade ZPC particles were used to remove pollutants such as SO_4_^2-^, Cr(VI), total chromium and F^-^ in groundwater from a mining area. The particles could effectively simultaneously remove chromium and fluorine ions. The static test results showed that the optimal reaction conditions were as follows: the volume ratio of Citrobacter was 35%, the dosage of hybrid material was 300 mL, and the reaction temperature was 35°C. The removal rates of SO_4_^2-^, Cr(VI), total chromium and F^-^ were 70.5%, 100%, 100% and 93.3%, respectively, and the pH value increased from 4.6 to 8.07. SO_4_^2-^ and Cr(VI) were removed by Citrobacter reduction in the ZPC particles and adsorption by the ZP material. Cr(Ⅲ) was removed by Citrobacter in the ZPC particles, which produced alkalinity, increased the pH value of the solution to generate Cr(OH)_3_ precipitation and was adsorbed by the ZP material.

The dynamic test results showed that the dynamic system with ZPC particles as the reaction layer had a better pollutant removal effect than the system with film-coated Citrobacter, and the removal effect was more stable. An influent hydraulic load of 3.0 m^3^/(m^2^·d) was the most appropriate. The selectivity of ZPC particles to various anionic pollutants was different, and the adsorption selectivity was in the order F^-^ > Cr(VI) > SO_4_^2-^.

The removal mechanism of pollutant removal by ZPC particles was further explained by SEM, FTIR and XRD. The Citrobacter has a strong reducing ability, reducing SO_4_^2-^ to S^2-^, Cr(VI) to Cr(III), and ZP material can adsorb F^-^, Cr(Ⅲ) and part of Cr(VI). The ZPC particles formed by intercalation can simultaneously remove SO_4_^2-^, Cr(VI), total chromium and F^-^. Therefore, this study offers new opportunities to investigate feasible and economic methods for SO_4_^2-^, Cr(VI), total chromium and F^-^ remediation from wastewater. Further process validation for large-scale applications is suggested as a further study to confirm the techno-feasibility of actual engineering applications.
